# Electronic Health Records for Predicting Outcomes to Work-Related Musculoskeletal Disorders: A Scoping Review

**DOI:** 10.1007/s10926-024-10175-1

**Published:** 2024-03-27

**Authors:** M. Wassell, A. Vitiello, K. Butler-Henderson, K. Verspoor, P. McCann, H. Pollard

**Affiliations:** 1 School of Computing Technologies, RMIT University, Melbourne, Australia; 2 School of Health, Medical and Applied Sciences, Central Queensland University, Queensland, Australia; 3 STEM Health and Biomedical Sciences, RMIT University, Melbourne, Australia; 4 Faculty of Health Sciences, Durban University of Technology, Durban, South Africa

**Keywords:** Electronic health records, Occupational injuries, Medical informatics, Occupational health physicians, Rehabilitation

## Abstract

**Purpose:**

Through electronic health records (EHRs), musculoskeletal (MSK) therapists such as chiropractors and physical therapists, as well as occupational medicine physicians could collect data on many variables that can be traditionally challenging to collect in managing work-related musculoskeletal disorders (WMSDs). The review’s objectives were to explore the extent of research using EHRs in predicting outcomes of WMSDs by MSK therapists.

**Method:**

A systematic search was conducted in Medline, PubMed, CINAHL, and Embase. Grey literature was searched. 2156 unique papers were retrieved, of which 38 were included. Three themes were explored, the use of EHRs to predict outcomes to WMSDs, data sources for predicting outcomes to WMSDs, and adoption of standardised information for managing WMSDs.

**Results:**

Predicting outcomes of all MSK disorders using EHRs has been researched in 6 studies, with only 3 focusing on MSK therapists and 4 addressing WMSDs. Similar to all secondary data source research, the challenges include data quality, missing data and unstructured data. There is not yet a standardised or minimum set of data that has been defined for MSK therapists to collect when managing WMSD. Further work based on existing frameworks is required to reduce the documentation burden and increase usability.

**Conclusion:**

The review outlines the limited research on using EHRs to predict outcomes of WMSDs. It highlights the need for EHR design to address data quality issues and develop a standardised data set in occupational healthcare that includes known factors that potentially predict outcomes to help regulators, research efforts, and practitioners make better informed clinical decisions.

**Supplementary Information:**

The online version contains supplementary material available at 10.1007/s10926-024-10175-1.

## Introduction

Work-related musculoskeletal disorders (WMSDs) are costly to governments, employers, injured employees, and the wider community. In 2012–13, WMSDs cost the Australian economy 28.2 billion dollars, accounting for 1.9% of the gross domestic product. More than one-third of the costs to the Australian economy are made up of manual handling cases [1], such as those coming from repetition, postural demands, and overuse type of movements. Serious workers’ compensation claims are claims that result in total incapacity from work for one week or more. Whilst serious claim numbers have reduced in Australia by 13% from 2001 to 2020, the median time lost from work due to WMSD injuries has increased from 5.0 to 5.2 weeks[2]. Globally, there are 26.44 million global disability adjusted life years (DALYs) caused by work-related injuries.

Musculoskeletal disorders include “a wide range of inflammatory and degenerative conditions affecting the muscles, tendons, ligaments, joints, peripheral nerves and supporting blood vessels.”[2, 3] Work-related musculoskeletal disorders encompass a broad group of conditions that can be multifactorial in its development and nature and may be associated with various physical, health and psychosocial workplace or personal life hazards. They often require multiple interventions to assist in their recovery, such as linking clinical care to workplace support mechanisms.

Addressing the problem of outcome prediction in WMSDs is common [4–8]. Outcomes that are typically studied include return to work, return to full duties, work ability and increasingly, stay at work. Much of the research and data collection related to outcome predictions in WMSDs is conducted through workers’ compensation claims database analysis [9]. A challenge with this type of research is that workers’ compensation claims databases only collect a few of the factors that have been shown to be potentially predictive of outcomes. Many other studies in this space are retrospective studies conducted via patient interviews [10] or utilising patient questionnaires [11].

Employees with a WMSD may choose to visit a range of therapists to help deal with their conditions, from emergency physicians, primary care practitioners, occupational physicians and by musculoskeletal (MSK) therapists, namely chiropractors, physiotherapists and osteopaths amongst others. Work-related musculoskeletal disorders are often managed by a range of practitioners working in various settings, from those in hospital systems, private clinics, and workplaces. A less studied area in this field is predicting outcomes of WMSD utilizing data collected by MSK therapists.

MSK therapists working in occupational health clinics or onsite at workplaces could collect many of the variables that are potentially predictive of outcomes to WMSD. Such variables can be challenging to collect through claims databases as they are not required for the primary purpose of the claims database. MSK therapists in occupational health clinics are often integrated within the workplace, which means they have may access to information on workplace culture, available workplace modifications, and job demands. These factors have all have been shown to be important for effective recovery from WMSDs [3] and thus are factors those clinicians should be determining from their patients, to help with good clinical decision making. Often MSK therapists' patient notes are still paper based, lacking the adoption of electronic health records (EHRs) that has been seen in primary and hospital-based systems. MSK therapists EHRs are primarily made up of unstructured boxes to collect data, with only some software yet to utilise structured data.

EHRs are a digital version of patients clinical notes and reports that are collected from patients, clinicians, laboratory or imaging providers, and are thus rich repositories of patient data and a potential source of clinical data for secondary use in predictive models and clinical decision support [12]. Therefore, EHRs are a possible solution to current challenges. Clinical registries or health databases, such as an occupational health registries often include EHR data but may include data from multiple sources. EHR use is increasing [13]. The WHO has listed several advantages of EHRs [14], including that they improve the quality of care, accuracy, reliability, and timeliness of patient information, providing insights into healthcare costs and outcomes. Analysis of EHR data has value in improving quality and reducing costs in healthcare in hospitals [15]. Despite the increasing use, there is a lack of direct insight into how or whether EHRs are currently being used for research into WMSDs. A scoping review is required to identify opportunities to move forward in the secondary use of EHR data for outcomes predictions research in WMSD. There is a need to synthesize existing literature to uncover best practices to help avoid building disparate EHR systems, losing opportunities to advance coherently in identifying predictors and reducing WMSD costs. To synthesize the literature, the review's objectives are to explore the extent of the research and identify gaps in the literature on the use of electronic health records used by MSK therapists in predicting outcomes for WMSDs. Specific themes of interest include identifying information sources relevant to predicting outcomes to WMSDs as well as the extent that standardised information has been used by MSK therapists when collecting WMSD information.

## Methodology

### Methodology Overview

The methodology was guided by established frameworks from Arksey and O’Malley [16], and the Peters et al. Preferred Reporting Items for Systematic Reviews and Meta-Analyses extension for Scoping Reviews (PRISMA-ScR) [17]. These frameworks enabled a comprehensive search of existing literature to identify articles related to the use of EHRs for predicting outcomes to WMSDs. The literature was summarised, and gaps were identified.s

### Search Strategy

The search strategy was developed by three researchers in consultation with two senior librarians. Key search terms included: workplace and musculoskeletal disorders, work related musculoskeletal disorders, musculoskeletal practitioners—physiotherapists, chiropractors, osteopaths, electronic health records, standard terminology, minimum data set, prognosis, predict, outcomes. Terms were searched in a variety of broad combinations to ensure a thorough search. A full list of search terms appears in appendix 3.

The search period was from January 1998 to April 2023 as EHRs have only been in mainstream existence for this time. Studies were limited to the English language due to resource constraints. A systematic search was conducted of the relevant databases, Medline, PubMed, CINAHL, and Embase. Hand-searching of reference lists and web-based searches for grey literature were also conducted. A secondary search of the literature using the same search strategy was conducted prior to submission in April 2023, to ensure the review was up to date.

### Study Selection and Sources of Evidence

A scoping review approach was selected to allow for synthesis without exclusion based on study types or formal assessment of study quality. This included policy and government documents. Three independent reviewers performed the title/abstract review. A consistency check was performed to determine the alignment of criteria prior to the full title/abstract screening, which resulted in minor changes to the criteria to provide clarity to definitions. For the full text assessment and data extraction, Covidence software (Veritas Health Innovation, Melbourne, Australia) was used. Two independent reviewers conducted the full-text search and data extraction. Inconsistencies were reviewed and resolved through discussion between the reviewers, with the lead author having the final decision.

### Data Extraction

A deductive approach to thematic analysis was utilised. Themes were used to develop the data chart for the process of data extraction. Extraction was performed by two reviewers independently. Inconsistencies were discussed between reviewers to come to the final decision. The data chart included all data recommended by the JBI Manual for Evidence Synthesis [18]. As well as this data were extracted based on the themes and included the use of EHRs, physical setting, type of practitioner, outcome measures, data sources, challenges and benefits of particular data sources, data quality issues, standardised information for data collection and potential predictors that could be captured in EHRs. Most data were summarised using free text analysis.

## Results

**Fig. 1:**
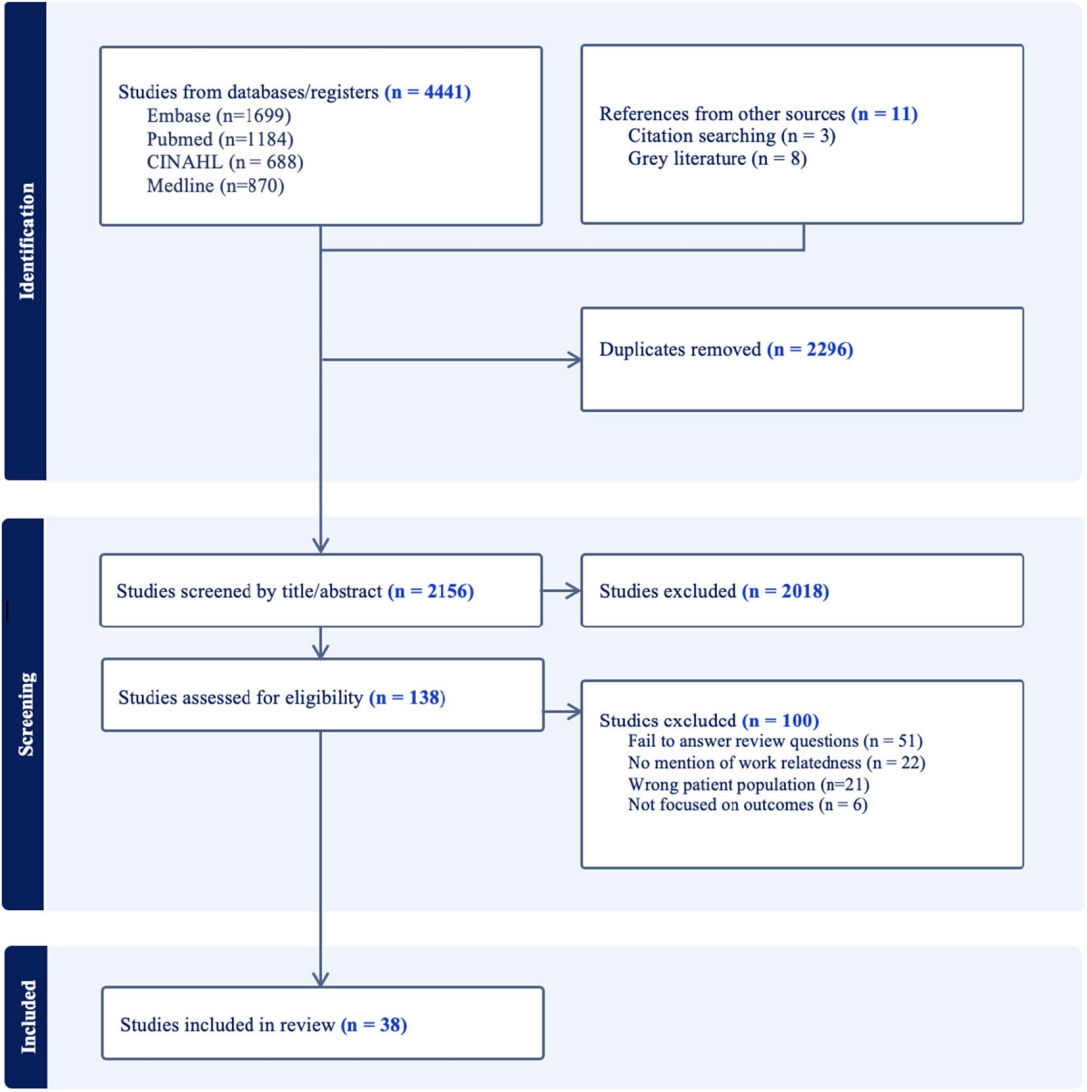
PRISMA diagram of literature search results

A total of 2156 unique papers were identified for title and abstract screening. Full text review of 138 papers resulted in 38 that met the broad inclusion criteria as shown in Fig. 1.

Overall, 3 themes were explored. The initial review objective was the use of electronic health records to predict outcomes WMSDs by MSK therapists. As very few papers were identified that fit this criterion, the first theme was modified. Theme 1 was the use of electronic health records for outcome prediction of WMSDs. Theme 2 is the sources of information used for predicting outcomes of WMSDs. Theme 3 is the adoption of standardised information for documenting WMSDs by MSK therapists when managing WMSDs.

### Methodological Quality of Articles

Of the 38 included papers, there were 18 cohort studies, 5 guidelines or government documents, 4 systematic reviews, 1 meta-analysis, 1 RCT, and the remaining were lower levels of evidence such as editorials and consensus articles. All government documents and guidelines were found in grey literature searches.

### Characteristics of Articles

Studies were conducted in 8 countries. 17 from the USA, 9 from Canada, 4 from Australia, 2 from the UK, Netherlands and Germany and 1 from China and Denmark. Included studies were from the years 2000 to 2022.

**Fig. 2:**
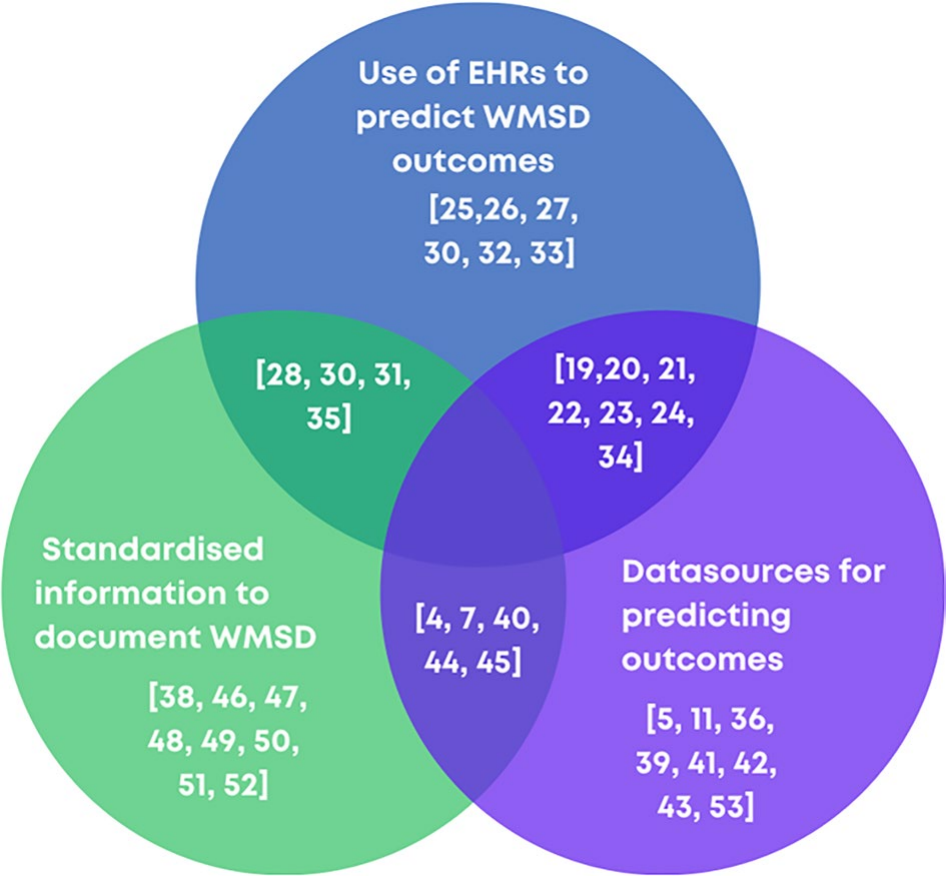
Themes represented in included articles

Figure 2 outlines the themes represented in each of the included papers.

There were 17 studies that were identified that assist in understanding the use of EHRs in predicting outcomes of WMSD. As these papers are the most relevant, their characteristics are described in Table 1.

**Table 1:** Papers relating to the use of EHRs in predicting outcomes to WMSDs

Study & year	Study design	Country	Objective	EHR study	WMSD specific	Outcome prediction	Data source(s)		Population	Practitioner setting
Razmjou 2021 [19]	Cohort	Canada	Explore characteristics of workers with multiple psychosocial flags and examine the relationship between them	x – electronic file	x	x	Electronic Patient File	NA	Cervical spine patients on a claim	Orthopeadic surgeon and physical therapist
Razmjou 2018 [20]	Cohort	Canada	Examine prevalence of psychological flags in workers with low back injuries, examine relationship between signs and ability to work	x – electronic file	x	x	Electronic patient file	NA	Low back injuries in patients on a claim	Orthopeadic surgeon and physical therapist
Cohen, 2009 [21]	Cohort	USA	Identify variables associated with return to unit in soldiers with back pain	x	x	x	EHR	Registry data	Back pain	Occupational physicians
Hiebert, 2012 [22]	Cohort	USA	Identify factors predictive of work duty status after LBP	x	x	x	EHR	PROMs—electronic	Back pain	Occupational physicians
Maeng, 2015 [23]	Cohort	USA	Explore feasibility of developing a dynamic predictive model using EHR data to identify high-cost patients	x	WMSDs within population	x	EHR	Insurance data	Low back pain	Primary care practitioners
George, 2021 [24]	Cohort	USA	Characterise patterns of recurrent care seeking and develop prediction model	x	WMSDs within population	x	EHR	Registry data	All MSDs	Physical therapists
Raja, 2017 [25]	RCT	USA	Pilot study developing RTW guidelines in EMR integrated tool	x	x		EHR	NA	Low back pain	Primary care practitioners
Middleton, 2020 [26]	Cohort	UK	Evaluate risk stratification and referral of high risk patients and identify the barriers and facilitators	x			EHR	PROMs—electronic	Low back pain	Primary care practitioners
Owens, 2021 [27]	Cohort	USA	Explore feasibility of EHR data for use in practice based research	x			EHR	NA	All recorded in EHR	Chiropractors
APHA, 2012 [28]	Guidelines	USA	Outline recommendations for incorporating occupational health information in EHRs				NA	NA	All recorded in EHR	NA
FDA, 2018 [29]	Guidelines	USA	Provide guidance on the use of EHR data in clinical investigations				NA	NA	All recorded in EHR	NA
vanTrijffel, 2019 [30]	Editorial	Nederlands	Discuss opportunities and limitations of using routinely collected data in research				NA	NA	All recorded in physiotherapy practice	Physiotherapists
Socias, 2016 [31]	Cohort	USA	Describe the demographics and baseline clinical indicators of migrant and seasonal farmworker patients	x	x		EHR	NA	Multiple health and MSD conditions	Primary care practitioners
Hill, 2014 [32]	Dissertation	USA	Explore factors associated with work-related injury in low socioeconomic status workers	x	x		EHR	PROMS—paper	Occupational injuries	Primary care practitioners
Husselbee, 2022 [33]	Cohort	UK	Implement and evaluate MSK PROMs in an EHR	x			EHR	PROMs—electronic	All MSD	Physiotherapists
Vallmuur, 2015 [34]	Systematic review	Australia	Synthesise research on machine learning approaches to textual injury surveillance data		x		WC claims data	Registry data	Occupational injuries	NA
Scott 2015 [35]	Cohort	USA	Use narrative text and codes to identify logging or agricultural injuries to develop a surveillance system	x			WC claims data, registry data	Hospital data and EHR data	Agricultural and logging injuries	Hospital

WC = Workers’ compensation

There were 18 papers related to the second theme, sources of data used in studies predicting outcomes to WMSDs, that were included in the review, the details of these papers are summarised in supplementary material 1. All papers except the systematic reviews listed the data sources used for the studies. Seven of the papers utilised workers’ compensation databases, 6 utilised occupational data registries, 6 utilised clinical/other registries, 6 utilised EHRs, 5 used PROMs or patient questionnaires, and the remaining data sources were patient interviews and a focus group.

Seventeen papers were identified that helped determine if there is a standard set of information that should be collected by MSK therapists when managing WMSDs. The characteristics of these papers are found in supplementary material 2. The discussion of the International Classification of Functioning, Disability and Health (ICF) core sets and instruments was found in 4 papers, 4 papers address coding systems such as the International Classification of Diseases (ICD) and the remaining papers report on limited consensus or are calls to include standard data or study potential predictors.

### Synthesis of Results

#### The Use of EHRs to Predict Outcomes to WMSDs

There is minimal research on the use of EHRs to predict outcomes of WMSDs with only two studies designed specifically using EHRs to predict outcomes of WMSD using MSK therapists for care [19, 20]. Through assessing a broader range of studies in this scoping review, the critical factors and feasibility of future use of EHRs to predict outcomes of WMSD can be reported.

In the studies specifically using EHRs by MSK therapists to predict outcomes to WMSDs, psychosocial factors are collected utilizing the ‘flag’ system [19]. This system identifies categories of psychosocial behaviours and beliefs, work beliefs and systemic factors that may influence recovery. The method of recording data were structured (e.g. yes/no response) and this was found to be beneficial in determining that the number of psychological factors present in a patient was related to work status.

Two other papers found it was feasible to predict the outcome of work status through EHR data. Both studies also highlight the importance of psychological factors such as psychiatric illness and fear avoidance beliefs in outcomes of WMSD [21, 22], which are commonly understood to be important risk factors for poor outcomes [5, 36] As this information is key for clinicians to understand their patients, psychosocial data within the EHR should be collected.

EHR data from physical therapists has been used to develop a predictive model for recurrent physical therapy care seeking by patients [24]. It has also been found feasible to develop a dynamic predictive model to determine the likelihood of high-cost outcomes of LBP patients based on EHR data [23]. Low back pain is a major component of WMSDs [37] and therefore it is relevant to consider low back pain studies that include work-related disorders. The opportunity of dynamic modelling is that it can be embedded into the EHR to provide decision support throughout patient care, for example, supporting different predictions at the first or third visit [23]. Whilst these models do not specifically focus on WMSD, they are relevant to outcomes in the WMSD population. Potentially, these outcomes have not been studied in the WMSD population as the required variables are not available in current data collection methods. Claims databases often don't capture the number of visits to MSK practitioners, comorbidities, lifestyle factors, psychosocial factors, practitioner characteristics and care plan details [38], as found in the models described [23, 24].

In further work, it has been found possible to utilise a workplace EHR for describing injuries in a worker population [31]. Many factors such as comorbidities, psychosocial and clinical factors that have been found relevant for describing injuries are the same as those that could be used for predicting outcomes in WMSDs.

#### Sources of Data for Predicting Outcomes to WMSDs

The studies that met the inclusion criteria are only a sample of the studies that look at predicting outcomes to WMSDs. A range of data sources were demonstrated, with workers' compensation claims databases being the most common data source for predicting outcomes to WMSDs.

In the relatively new area of research of predicting outcomes to WMSD using EHRs, it is prudent to determine the challenges with existing data sources, to ensure that effective EHR systems can be built for future research. Each data source has its strengths and weaknesses. Claims databases benefit from large number of records for available analysis [39, 40] as do EHRs.

One of the biggest challenges of using EHRs as a data source for research is data quality. Some aspects include missing data, unstructured data, interoperability issues between datasets and collection of the right data. Missing data is a challenge for many EHR studies [23], missingness of desirable research variables can be nearly half what is required [27, 31]. Missing data is not, however, only a challenge for EHRs, and exists in claims databases [8, 34, 38, 41] as well as in studies utilising patient questionnaires or PROMs [42, 43]. Missing data can also be reported due to issues in coding unstructured data, due to misspellings or abbreviations but there is the potential for this to occur in EHRs [21, 31], and also in claims databases [34].

Unstructured data analysis is reported as challenging in EHR datasets [31] and claims databases [34], which is due to processing textual data [34] and coding the data to recognised coding formats such as the ICD, leading to potentially lower quality analysis or the need to exclude records [31, 35, 39]. A study found that many variables that were available for analysis, such as medication lists, education and income that were excluded from the study due to inabilities to process unstructured data [31]. Unstructured data also has its benefits, such as being able to capture sequence of events that can be missed from structured data [35].

There have been calls for utilising structured data in EHRs where possible. The benefits are demonstrated by Razmjou, finding important predictors of outcomes to WMSDs within structured psychosocial data [19, 20].

Unstructured data leads to issues with the time taken for analysis in EHR datasets [26, 27]. Some authors have even found the time for analysis of unstructured data unreasonable for the study [27]. However, whilst time-consuming, processing unstructured data can provide important insights as it has been found necessary for some studies, as EHR systems may not contain the necessary coding to identify injuries, such as logging industry injuries [35]. Similar time-related issues for unstructured data analysis also exist in analysing claims forms for claims databases or registry data [34] and data analysis in paper-based systems for patient questionnaires or patient-reported outcome measures (PROMs). An example of how structured electronic data can influence time for analysis was found by a study converting PROMs to electronic format, allowing analysis of 18 months of validated PROMs data in 2 days [33].

One of the primary use cases for EHRs is to assist clinicians to make effective clinical decisions. Many authors report on the challenge of using secondary data for research when conducting EHR studies. Desirable variables for analysis may not be available. [21, 24, 27, 35, 44] or if they are, there is variability between practitioner collection methods [31]. Again, this is not an issue unique to EHR studies. Researchers utilising claims databases suffer the same challenge[8, 34] and overcome this issue by linking data sources [8, 11, 21, 23, 26, 34, 39], which can introduce quality issues such as errors in data translation.

#### Adoption of Standardised Information for Documenting WMSDs by MSK Therapists when Managing WMSDs

The scoping review identified no standardised set of information that MSK practitioners should collect when managing WMSDs or that which should be collected to assist in predicting outcomes of WMSDs. There is little consensus on what variables should be routinely collected for workers in occupational health clinics or primary care [45]. However, calls have been made for better occupational health information to be added to standard data capture. In the USA in 2011, the Institute of Medicine recommended including occupation and industry data in EHRs, to improve diagnosis, develop more focused treatment plans and streamline return-to-work guidance [28, 31]. Cancelliere, in their systematic review, reports on an improved set of return-to-work principles that can be used as guidance for practitioners when questioning patients with WMSD [4].

National coding sets for countries exist for coding information, such as the Type of Occurrence Classification system (TOOCS) in Australia [46] and the Occupational Injury and Illness Classification System (OIICS) in the USA. In different manners, both systems code the nature of injury/disease, body location, mechanism/event and the source/agency of the injury/disease. These codes are relevant for MSK practitioners to use in EHRs when managing WMSD. Australia has a required national data set for workers’ compensation claims [38]. Treating practitioners must capture specific information, such as diagnosis and mechanism of injury, in free text form, which is then coded using a national coding set. The USA does not yet have standard data collection between all jurisdictions for workers’ compensation records [47].

The ICF is a framework for a systematic approach to outcome measurement that has been widely adopted within rehabilitation fields [48]. A systematic review found a comprehensive core set of questionnaires relevant to work-related rehabilitation contained 90 domains from the ICF and a brief set contained 13 items. [49]. In related work, a core set of measures for vocational rehabilitation in MSK pain was developed that consists of 18 domains measured with 12 instruments [50]. Whilst comprehensive, these sets require users to be able to identify the specific aspect of work functioning that should be measured [49]. The collection of data relating to work activity and participation has been advocated for [48] whilst a study assessing the item bank for work participation found 122 relevant questions for employees with MSDs [51].

Furthermore, the ICD is often used to code morbidity and mortality data and is often integrated into EHRs [21, 31, 36, 52]. However, the coding system may need to be expanded for MSK therapists managing with WMSD. For example, ICD codes were found to be unavailable for identifying logging and agricultural injuries [35].

On top of the frameworks and coding systems, the factors that have been found to be predictive of outcomes could also be considered as relevant data collection by MSK therapists dealing with WMSDs to help practitioners make more informed decisions as a component in delivering evidence-based care.

Table 2 indicates the categories of variables that should be considered for collection by MSK therapists, as they have been shown to be potentially predictive of outcomes to WMSDs. Whilst not exhaustive due to the scope of the study, some examples are given.

**Table 2:** Categories of variables for collection by MSK therapists managing WMSDs

Body structure/function	Body structure/function examples	Personal	Personal examples	Environment	Environment examples	Activities and participation factors	Activities and participation factors examples
Intervention	Multidisciplinary [4], educational/psychological programs [4], exercise [4], type of intervention [4, 11]	Demographic [23]	Age [4, 19], gender [4, 19], occupation [4]	Workplace cultural/psychological factors [42, 53]	Workplace social support [4]	RTW activities	Stakeholder participation in RTW process [4], RTW coordination [4], early contact with worker [4]
Injury specific [11, 53]	Severity and location [11, 53]	Socioeconomic [11, 42]	Low personal income [4], education level [4]	Job physical demands [8]	Heavy physical workload [4, 8]	Previous History [8]	Multiple past claims [8], previous sick leave [4], high health care system interaction [8]
Clinical findings [42]	Pain score [4, 19], night sweats [24]	Lifestyle [42]	Alcohol intake [4]	Job psychological demands	Locus of control over work [4]	System factors	Compensation level [4], work status [19]
Rehabilitation programs [43]	Completion of prescribed program [43]	Medical history [43]	Perceived general health [42], comorbidities [4]	Social support	Family/community support [4]	Activities	Activity limitation or restriction of participation [4, 20], work accommodation [4, 53], health locus of control [19]
		Psychological (orange flags) [5, 22, 42]	Anxiety or depression [5, 22, 42]	Work environment			
		Psychosocial (yellow flags)	Fear avoidance behaviours, self-efficacy belief [4, 5], beliefs of works responsibility for the WMSD [42], stress [4], number of psychosocial flags [19, 20]				
		Work beliefs (blue flags)	Perceived work demands, [4], work satisfaction [4], beliefs of employees works responsibility for the WMSD [42], return to work expectations work [4, 5, 42, 45]				

A consideration in deciding what information to collect in an EHR must be the time taken to complete the documentation. Practitioners have a high documentation burden and limited time [26]. The core sets that have been reported in this section may be deemed to be too extensive for routine clinical use [50]. Time to complete documentation is a consideration not only for practitioners using EHRs but also for patients or employees completing questionnaires [50, 51].

## Discussion

This paper analyses the current published and grey literature relating to the use of EHRs in predicting outcomes of WMSDs. There are limited papers specifically addressing the use of EHRs to predict outcomes to WMSDs by MSK therapists [19, 20]. The most common data sources used to predict outcomes of WMSD are claims databases, clinical and occupational registries. There is no standard set of data collection that MSK practitioners should collect when treating WMSDs or to assist in predicting outcomes of WMSDs. There is limited consensus on what variables should be routinely collected for workers in occupational health clinics.

To understand the reasons for our limited findings, consideration should be given to the barriers to research in EHRs and how further work from a policy, awareness and research prospective may advance the following areas, including; interoperability of EHR systems, data standardisation, minimum data sets, integration of skills between clinicians and technical operators to develop and interpret data [34] and EHR vendors focused on broad applicability of their products and billing purposes rather than the needs of healthcare providers.

The implication of not systematically using EHRs or developing policy around minimum data variables collection is that countries may continue to struggle to improve the costs and outcomes of WMSD. There is an opportunity to address the challenges with predicting outcomes for WMSD as healthcare globally is shifting towards value-based care models. To provide value-based care, there must be a measurement of outcomes. Collecting outcomes data through digital pathways such as electronic health records (EHRs) is therefore becoming more important. Due to this and the authors’ observations that MSK therapists are increasingly working in occupational health clinics, research on MSK therapists' outcomes for WMSDs will likely become more critical. EHRs will allow further study of clinical factors and treatment effects in WMSDs as there are currently gaps in knowledge by failing to understand the specifics of MSK care [9, 41] as details are not available for analysis [9].

The challenges relating to data quality in EHRs for reuse are being increasingly studied, and frameworks specific for assessing EHR data quality have been established [54–56], which can be used as a guide to develop EHRs. In designing EHRs for secondary use, key information needs to be collected and in the right format. The intrinsic structure of the data, such as the conformance, completeness and plausibility must be considered. As well, contextual factors such as the relevance, timeliness, user interface, accessibility, reusability and governance over the data collection, management, and operational use needs consideration. Further, the technical considerations such as the operating platform, security and interoperability are all key parameters to determine if EHR data can be used for research predicting outcomes to WMSD. There are benefits and challenges of structured and unstructured data collection, and balancing data collection with time considerations and usability of the EHR system [57].

In discussing the challenge of data quality within EHRs it can be observed that the included studies relating to predicting outcomes rarely address data quality. Half of the papers address how missing data is managed whilst just three papers address other aspects of data quality, such as those mentioned in the paragraph above. As McIntosh identified in 2000, the control of data collected in claims database type studies is beyond the investigators control [41]. Data quality issues from claims databases can come from coding and categorising free text physician diagnosis or a variation in physician understanding of the necessary data capture fields definitions [45]. Additionally, data collection techniques and the setting may influence patient responses to questionnaires, such as in the case of collecting psychosocial data in a workplace where patients may not feel safe to answer truthfully. More data quality issues are identified in the studies that utilise EHRs as a data source than those using other sources for secondary research. Integration of the knowledge on data quality in the health informatics field may benefit occupational health research that could potentially improve the quality of studies addressing predictors to outcomes in WMSD. Further understanding of issues relating to data quality may help data collectors understand how to avoid potential quality issues that could improve study results.

The findings of this study outline the range of coding systems and frameworks used to record information about WMSD with a vast number of instruments and questions, creating a challenge for practitioners to know the most appropriate measures to use and when to use them. As EHRs become more prevalent, there is a significant opportunity for regulatory bodies, such as Workers’ Compensation authorities, to require that practitioners collect variables that are valuable in predicting outcomes. This would assist in ensuring EHR systems are designed in coming years that support standard data collection, in line with increasing regulations around interoperability.

As EHR system adoption is growing, it is vital that a minimum or standard set of variables for collection by MSK practitioners dealing with WMSD is developed. There must be agreement on the key information to collect and standardised formats, which utilise both structured and unstructured data. Creating a minimum dataset is vital work to assist practitioners in minimising time spent on documentation. Currently, the documentation burden from utilising ICF core sets is prohibitive. Determining single-question measures, such as the Work Ability Index [58], is required to ensure clinicians can capture all the information required to make high-quality clinical decisions without a high documentation burden. This, in turn, can lead to higher-quality EHR data for research.

Musculoskeletal therapists who deal with WMSDs are suitably placed to collect all the relevant information through EHRs. Factors that have been shown to be predictive of outcomes are important for clinicians to collect as part of evidence-based practice, to improve their clinical outcomes, whilst also creating the potential for population-level improvement of outcomes through secondary EHR data use. There is real potential for EHRs to increasingly contribute to real-world evidence [30] and reduce WMSD costs. MSK therapists and occupational therapists may also be able to collect more accurate return to work details, as currently, surrogate measures are used, such as cessation of claims payments [41].

The collection of the right information within the EHR, in the right formats will provide opportunities for dynamic clinical decision support tools [44, 59], such as RTW tools, that can assist practitioners and RTW providers in providing recommendations to patients with WMSD [25]. Clinical decision support that is integrated into an EHR could improve compliance to guidelines, potentially improving clinical outcomes and reducing RTW costs.

This scoping review has reviewed a broad scope of papers within the peer-reviewed and grey literature to assess the current state of research. This paper successfully highlights the significant gaps that still exist in the literature to develop our use of EHRs by MSK practitioners to assist with WMSDs. The review highlights the need for work to be done to standardise clinical questions to ensure practitioners are asking the right questions in the right format, to minimise their time taken within the EHR and gain optimal value in their ability to make effective clinical decisions. These efforts will contribute meaningful data to potential research efforts to help not only their patients but WMSD patients as a whole, globally.

### Strengths and Limitations

This research has been able to capture a broad range of literature to allow determination of the current state of research in the use of EHRs to predict outcomes to WMSD. Additionally outlining the gaps in this area can help determine what is required to allow EHRs in the future to successfully predict outcomes to WMSDs.

A limitation of the study is that papers in languages other than English grey literature may have been missed. For example, many of the government documents found through grey literature searching are likely to have similar documents available across many different countries. As there are so few studies in the field, there is a small number of data points, that may be interpreted as unrepresentative of the total.

Additionally, a specific limitation of this study was achieving the correct depth and of breadth of analysis. This has been reported as a challenge in scoping reviews in general [60]. For this study, the interest was in EHRs that MSK therapists used, but studies were excluded that focused on traumatic injuries that MSK therapists wouldn’t typically manage. There may be more literature in this sector that could help guide the discussion on the use of EHRs in predicting outcomes of WMSD, even if this information is in a slightly different field.

Finally, individual outcomes were not listed in the search strategy, however, by using MeSH and subject heading terms the authors believe a broad range of outcomes have been included.

### Future research

Future research is needed to develop standard and minimum datasets, specifically for MSK therapists and physicians working in occupational settings. Further studies must address data quality within EHRs to determine if the quality is sufficient that they can be used to predict outcomes to WMSDs.

## Conclusion

The review outlines the limited research on using EHRs to predict outcomes of WMSDs and highlights the need for a standardized data set in occupational healthcare. EHR design for practitioners managing WMSDs should address data quality issues, to improve the data value, interoperability and documentation burden.

To help practitioners make informed clinical decisions, factors that predict WMSD outcomes are crucial to collect. As such, occupational MSK therapists are well-suited to collect the relevant variables identified in the study, including injury specific factors, workplace organisational and cultural factors, job physical, psychological and health demands, social factors, clinical findings and intervention-based factors. By incorporating these factors into EHR workflows, the data is likely to be valuable in predicting outcomes of WMSD to assist in reducing the growing costs of WMSDs, as well as in helping practitioners make more informed clinical decisions about patient care.

The findings offer guidance to regulators, healthcare practitioners and other stakeholders in EHR workflow development and data collection as it outlines key considerations in collecting clinical data in EHRs for research.

## Supplementary Information

Below is the link to the electronic supplementary material.

Supplementary file1 (DOCX 53 kb)

## Data Availability

The datasets generated during and/or analysed during the current study are available from the corresponding author on reasonable request.
