# Supplementary material for: Electronic Health Records for Predicting Outcomes to Work-Related Musculoskeletal Disorders: A Scoping Review
Source: J Occup Rehabil. 2024 Mar 27;34(4):770–82. doi: 10.1007/s10926-024-10175-1 (PMC11550283; doi:10.1007/s10926-024-10175-1)
Supplement: Supplementary file 1 — Supplementary file1 (DOCX 53 kb) [file 10926_2024_10175_MOESM1_ESM.docx]

# SUPPLEMENTARY INFORMATION

## Supplementary Material 1. Paper characteristics - Data sources used in included studies predicting outcomes to WMSDs.

| **Author & year** | **Study design** | **Country** | **Population** | **Data source(s)** | |
| --- | --- | --- | --- | --- | --- |
| Sullivan 2022 [40] | Meta analysis | Canada | Low back pain in workers | Occupational rehab clinic | WC claims database |
| Sears 2014 [29] | Cohort | USA | All work-related conditions presenting to a hospital | WC claims database | Clinical trauma registry |
| Sears 2013 [29] | Cohort | USA | All work-related conditions presenting to a hospital | WC claims database | Clinical trauma registry |
| Proctor 2005 [35] | Cohort | USA | Patients with chronic disabling occupational MSK disorders | PROMS-Paper | Telephone interview |
| Maeng 2015 [25] | Cohort | USA | Low back pain patients presenting to primary care | EHR | WC claim database & hospital billing data |
| Jensen 2013 [34] | Cohort | Denmark | Low back pain patients on sick leave | WC claims database | Prospective patient interview |
| Hu 2014 [11] | Cohort | China | Work related hand injuries in hospitals | Prospective & retrospective patient interview | PROMS - paper |
| Hiebert 2012 [22] | Cohort | USA | Navy and marine officers with low back pain seeking care from emergency or primary care clinics | EHR | PROMS - paper |
| Hayden 2019 [5] | Systematic review | Canada | Low back pain from any setting | Data sources not reported | - |
| Gross 2013 [39] | Cohort | Canada | All work-related conditions in patients receiving workers' compensation | WC claims database | Occupational rehab Registry |
| Gross 2005 [8] | Cohort | Canada | Low back pain in workers' compensation patients | WC claims database | Occupational rehab registry |
| George 2021 [24] | Cohort | USA | All musculoskeletal conditions presenting for physical therapy | EHR | Clinical registry |
| Dionne 2013 [49] | Qualitative research | Canada | Workers with low back pain currently off work | Retrospective patient interview | Focus groups |
| Cohen 2009 [21] | Cohort | Australia | Low back pain in soldiers who are not at work | EHR | Occupational injury (military) registry |
| Cohen 2010 [23] | Cohort | USA | All work-related conditions in service members who had time off work/away from unit | Occupational Injury (military) registry | - |
| Cancelliere 2016 [4] | Systematic review | USA | All health and injury conditions in workers who have had time off work | Data sources not reported | - |
| Razmjou 2022 [19] | Cohort | Canada | Cervical spine patients on a workers' compensation claim | Electronic Patient File | - |
| Razmjou 2018 [20] | Cohort | Canada | Low back injuries in patients on a workers' compensation claim | Electronic Patient File | - |

## Supplementary Material 2. Paper characteristics – Standard data sets for use in predicting outcomes to WMSDs.

| **Author & year** | **Study design** | **Country** | **Objective** | **Population** |
| --- | --- | --- | --- | --- |
| Sullivan 2022 [40] | Meta analysis | Canada | Assess workers expectations for RTWs association with RTW, explore relationships and prognostic factors | Workers with low back pain |
| Sternberg 2018 [45] | Systematic review | Germany | Identify and describe questionnaires measuring work functioning that reference the ICF | Individuals with MSK disorders |
| Socias 2016 [28] | Cross sectional cohort | USA | To describe the demographics and baseline clinical indicators of patients in an EHR by worker and migrant status | Patients with all conditions presenting to health clinic |
| Reneman 2013 [46] | Consensus study | Nederlands | Develop a core set of measures specifically for vocational rehabilitation of patients with subacute and chronic MSK pain | Individuals with subacute and chronic MSK pain |
| Nyhus 2021 [48] | Opinion piece | USA | To explore commonly billed occupational injuries and illnesses. | Occupational injuries and illnesses |
| Mueller 2013 [47] | Consensus study | Germany | To develop s self assessment instrument and measure work performance in patients with MSK diseases | Patients with MSK disease |
| Hayden 2019 [5] | Systematic review | Canada | To synthesize evidence on association between expectation of recovery with disability outcomes | Adults with low back pain |
| Gross 2013 [39] | Cohort | Canada | To develop an algorithm and clinical decision support tool to help injured workers towards optimal rehabiliation interventions | All work-related conditions |
| NOHSC, 2004 [27] | Government document | Australia | To review data items and concepts contained in the national data set for compensation-based statistics, to assist in prevention of occupational injury and produce uniform national indicators | All work-related conditions |
| Cancelliere 2016 [4] | Systematic review | Canada | To identify prognostic factors for RTW across health and injury conditions and their association with RTW outcomes | Workers with health and injury conditions |
| APHA 2012 [41] | Government document | USA | To endorse a committee report and recommendations to capture patients work information into EHRs | Patients in the USA healthcare system |
| AlHeresh 2015 [44] | Review | USA | To develop a framework for work outcomes with individuals with chronic MSK conditions and to review existing frameworks | All work-related conditions |
| FDA, 2018 [59] | Government document | USA | To provide guidance on how to use EHRs as a source of data in clinical investigations regarding interoperability, quality and integrity of data | EHR data |
| NOISH, 2014 [43] | Government document | USA | To describe elements of the WC program in the US and the potential to utilize records for public health purposes | WC claims |
| ASCCC, 2008 [42] | Government document | Australia | To list and describe the coding set to improve the quality of the National Data Set for work-related conditions | All work-related conditions |
| Razmjou 2022 [19] | Cohort | Canada | Explore characteristics of workers with multiple psychosocial flags and examine the relationship between them | Cervical spine patients on a WC claim |
| Razmjou 2018 [20] | Cohort | Canada | Examine prevalence of psychological flags in workers with low back injuries, examine relationship between signs and ability to work | Low back injuries in patients on a WC claim |

## Supplementary Material 3. PICO_1

The following PICO framework was assessed by the primary researcher and two senior librarians as appropriate.

| P 1a | Workplace (not applicable on its own, only with 1C) |
| --- | --- |
| P 1b | Musculoskeletal disorders (not applicable on its own, only with 1C) |
| P 1c | Work-related musculoskeletal disorders |
| P2 | Musculoskeletal practitioners |
| I1 | Electronic health records |
| I2 | Standard information |
| I3 | Datasource |
| Co1 | Outcome prediction |

**Supplementary Material 4. Search_Terms**

|  | **Embase** | **Pubmed** | **MEDLINE** | **CINAHL** |
| --- | --- | --- | --- | --- |
| Co | characteristic* | characteristic* | characteristic* | characteristic* |
| Co | prognos* | prognos* | prognos* | prognos* |
| Co |  | Prognosis(MeSH) | exp Prognosis/ |  |
| Co | predict* | predict* | predict* | predict* |
| Co | prediction'/exp |  |  |  |
| Co | clinical outcome*' | "clinical outcome*" | "clinical outcome*" | "clinical outcome*" |
| Co | clinical outcome'/exp |  |  |  |
| Co | treatment outcome*' | "treatment outcome*" | "treatment outcome*" | "treatment outcome*" |
| Co | treatment outcome'/exp | Treatment Outcome(MeSH) | exp Treatment Outcome/ |  |
| Co | outcome* | outcome* | outcome* | outcome* |
| Co | outcome'/exp | Outcome Assessment, Health Care(MeSH) | exp Outcome Assessment, Health Care/ |  |
| I | data OR dataset OR 'data base'/exp OR 'data source*' |  |  |  |
| I | data source*' | "data source*" | "data source*" | "data source*" |
| I | database | database* | database* | database |
| I | data base'/exp | Database [Publication Type] |  |  |
| I | dataset | dataset* | dataset* | dataset |
| I |  |  |  | (MH "Common Data Elements") OR (MH "International Classification of Diseases") OR (MH "International Classification of Functioning, Disability, and Health") OR (MH "Logical Observation Identifiers, Names and Codes") OR (MH "Snomed") |
| I | standardised terminolog*' | "standardised terminolog*" | "standardised terminolog*" | "standardised terminolog*" |
| I | common terminolog*' | "common terminolog*" | "common terminolog*" | "common terminolog*" |
| I | validated dataset*' | "validated dataset*" | "validated dataset*" | "validated dataset*" |
| I | validated question*' | "validated question*" | "validated question*" | "validated question*" |
| I | clinical data*' | "clinical data*" | "clinical data*" | "clinical data*" |
| I |  |  |  | "minimum data*" |
| I | minimum data*' | "minimum data*" | "minimum data*" | (MH "Health Plan Employer Data and Information Set") OR (MH "Outcome Assessment Information Set") |
| I | standard data collection' | "standard data collection" | "standard data collection" | "standard data collection" |
| I | standard dataset' | "standard dataset" | "standard dataset" | "standard dataset" |
| I | standard data' | "standard data" | "standard data" | "standard data" |
| I | emr*' | "emr*" | "emr*" | "emr*" |
| I | ehr*' | "ehr*" | "ehr*" | "ehr*" |
| I |  | Medical Records Systems, Computerized(MeSH) | exp Medical Records Systems, Computerized/ |  |
| I | patient medical record*' | "patient medical record*" | "patient medical record*" | "patient medical record*" |
| I | electronic patient record*' | "electronic patient record*" | "electronic patient record*" | "electronic patient record*" |
| I | electronic patient record'/exp |  |  |  |
| I | electronic medical record*' | "electronic medical record*" | "electronic medical record*" | "electronic medical record*" |
| I | electronic medical record'/exp |  |  |  |
| I | electronic health record*' | "electronic health record*" | "electronic health record*" | electronic health record*" |
| I | electronic health record'/exp | Electronic Health Records(MeSH) | exp Electronic Health Records/ | (MH "Electronic Health Records+") |
| P | manipulative medicine'/exp |  |  |  |
| P | physical rehabilitation' | "physical rehabilitation" | "physical rehabilitation" | "physical rehabilitation" |
| P | physical rehabilitation'/exp | Physical and Rehabilitation Medicine(MeSH) | exp "Physical and Rehabilitation Medicine"/ |  |
| P | manual therap*' | "manual therap*" | "manual therap*" | "manual therap*" |
| P |  | Musculoskeletal Manipulations(MeSH) |  |  |
| P | physical therap*' | "physical therap*" | "physical therap*" | physical therap*" |
| P |  | Physical Therapy Modalities(MeSH) | exp Physical Therapy Modalities/ | (MH "Physical Therapy+") |
| P |  | Physical Therapists(MeSH) | exp Physical Therapists/ |  |
| P | musculoskeletal therap*' |  |  |  |
| P | myotherap* |  |  |  |
| P |  | Osteopathic Physicians(MeSH) | exp Osteopathic Physicians/ | (MH "Osteopaths") |
| P | osteopath* | osteopath* | osteopath* | osteopath* |
| P | physiotherap* | physiotherap* | physiotherap* | physiotherap* |
| P | physiotherapy'/exp |  |  | chiropract* |
| P | chiropract* | chiropract* | chiropract* | (MH "Chiropractic+") |
| P |  | Chiropractic(MeSH) | exp Chiropractic/ | (MH "Chiropractic+") OR (MH "Chiropractic Practice") OR (MH "Chiropractic Assessment") |
| P | manual therapist'/exp |  |  | (MH "Manual Therapy+") |
| P |  |  |  |  |
| P | workplace injur*' | "workplace injur*" | "workplace injur*" | "workplace injur*" |
| P | work-related back pain*' | "work-related back pain*" | "work-related back pain*" | "work-related back pain*" |
| P | work-related musculoskeletal pain*' | "work-related musculoskeletal pain*" | "work-related musculoskeletal pain*" | "work-related musculoskeletal pain*" |
| P | workplace musculoskeletal pain*' | "workplace musculoskeletal pain*" | "workplace musculoskeletal pain*" | "workplace musculoskeletal pain*" |
| P | workplace musculoskeletal disorder*' | "workplace musculoskeletal disorder*" | "workplace musculoskeletal disorder*" | "workplace musculoskeletal disorder*" |
| P |  | "occupational injur*" | "occupational injur*" | "occupational injur*" |
| P | occupational injur*' | Occupational Injuries(MeSH) | exp Occupational Injuries/ | (MH "Occupational-Related Injuries") |
| P | occupational accident*' | "occupational accident*" | "occupational accident*" | "occupational accident*" |
| P | occupational accident'/exp | Accidents, Occupational(MeSH) | exp Accidents, Occupational/ | (MH "Accidents, Occupational+") |
| P | wmsds' | "wmsds" | "wmsds" | "wmsds" |
| P | wmsd' | "wmsd" | "wmsd" | "wmsd" |
| P | work-related musculoskeletal disorder*' | "work-related musculoskeletal disorder*" | "work-related musculoskeletal disorder*" | work-related musculoskeletal disorder*' |
| P | work related musculoskeletal disorder'/exp |  |  |  |
| P | neck pain' | "neck pain" | "neck pain" | (MH "Neck Pain") |
| P | upper limb disorder*' | "upper limb disorder*" | "upper limb disorder*" | "upper limb disorder*" |
| P | musculoskeletal pain' | "musculoskeletal pain" | "musculoskeletal pain" | "musculoskeletal pain" |
| P | musculoskeletal pain'/exp | Musculoskeletal Pain(MeSH) | exp Musculoskeletal Pain/ |  |
| P | back pain*' | "back pain*" | "back pain*" | "back pain*" |
| P |  | Back Pain(MeSH) | exp Back Pain/ |  |
| P | backache'/exp |  |  |  |
| P | musculoskeletal disorder*' | "musculoskeletal disorder*" | "musculoskeletal disorder*" | "musculoskeletal disorder*" |
| P | musculoskeletal disorders'/exp | Cumulative trauma disorders(MeSH) | exp Cumulative Trauma Disorders/ |  |
| P | work | work | work | work |
| P | work'/de | "Work"(MeSH) | exp Work/ | (MH "Work+") |
| P | workplace*' | "workplace*" | "workplace*" | "workplace*" |
| P | workplace'/exp | Workplace(MeSH) | exp Workplace/ | (MH "Work Environment+") |
| P | worker*' | worker* | worker* | worker* |
| P | worker'/exp |  |  |  |

**Supplementary Material 5_ 5 PUBMED_SEARCH_STRATEGY_SUMMARY**

| 1 | (("occupational groups"[MeSH Terms] OR ("occupational"[All Fields] AND "groups"[All Fields]) OR "occupational groups"[All Fields] OR "worker"[All Fields] OR "workers"[All Fields] OR "worker s"[All Fields] OR "workplace"[MeSH Terms] OR "workplace*"[All Fields] OR "Work"[MeSH Terms] OR ("Work"[MeSH Terms] OR "Work"[All Fields])) AND ("cumulative trauma disorders"[MeSH Terms] OR "musculoskeletal disorder*"[All Fields] OR "back pain"[MeSH Terms] OR "back pain*"[All Fields] OR "musculoskeletal pain"[MeSH Terms] OR "musculoskeletal pain"[All Fields] OR "upper limb disorder*"[All Fields] OR "neck pain"[All Fields])) OR ("work related musculoskeletal disorder*"[All Fields] OR "wmsd"[All Fields] OR "wmsds"[All Fields] OR "accidents, occupational"[MeSH Terms] OR "occupational accident*"[All Fields] OR "occupational injuries"[MeSH Terms] OR "occupational injur*"[All Fields] OR "workplace musculoskeletal disorder*"[All Fields] OR (("workplace"[MeSH Terms] OR "workplace"[All Fields] OR "workplaces"[All Fields] OR "workplace s"[All Fields]) AND ("musculoskeletal system"[MeSH Terms] OR ("musculoskeletal"[All Fields] AND "system"[All Fields]) OR "musculoskeletal system"[All Fields] OR "musculoskeletal"[All Fields]) AND "pain*"[All Fields]) OR "work related musculoskeletal pain*"[All Fields] OR "work related back pain*"[All Fields] OR "workplace injur*"[All Fields]) |
| --- | --- |
| 2 | "chiropractic"[MeSH Terms] OR "chiropract*"[All Fields] OR "physiotherap*"[All Fields] OR "osteopath*"[All Fields] OR "osteopathic physicians"[MeSH Terms] OR "physical therapists"[MeSH Terms] OR "physical therapy modalities"[MeSH Terms] OR "physical therap*"[All Fields] OR "musculoskeletal manipulations"[MeSH Terms] OR "manual therap*"[All Fields] OR "physical and rehabilitation medicine"[MeSH Terms] OR "physical rehabilitation"[All Fields] |
| 3 | "electronic health records"[MeSH Terms] OR "electronic health record*"[All Fields] OR "electronic medical record*"[All Fields] OR "electronic patient record*"[All Fields] OR "patient medical record*"[All Fields] OR "medical records systems, computerized"[MeSH Terms] OR "ehr"[All Fields] OR "emr"[All Fields] |
| 4 | "standard data"[All Fields] OR "standard dataset"[All Fields] OR "standard data collection"[All Fields] OR "minimum data*"[All Fields] OR "clinical data*"[All Fields] OR "validated question*"[All Fields] OR "validated dataset*"[All Fields] OR "common terminolog*"[All Fields] OR "standardised terminolog*"[All Fields] |
| 5 | "dataset"[Publication Type] OR "datasets as topic"[MeSH Terms] OR "dataset"[All Fields] OR "database"[All Fields] OR "database s"[All Fields] OR "databased"[All Fields] OR "databases"[All Fields] OR "databasing"[All Fields] OR "data source*"[All Fields] |
| 6 | "outcome assessment, health care"[MeSH Terms] OR "outcome*"[All Fields] OR "treatment outcome"[MeSH Terms] OR "treatment outcome*"[All Fields] OR "clinical outcome*"[All Fields] |
| 7 | "predict*"[All Fields] OR "prognosis"[MeSH Terms] OR "prognos*"[All Fields] OR "characteristic*"[All Fields] |
| 8 | #1 AND #2 AND #3 AND 6 AND #7 |
| 9 | #1 AND #2 AND #3 AND #7 |
| 10 | #1 AND #3 AND #6 AND #7 |
| 11 | #1 AND #3 AND #7 |
| 12 | #2 AND #3 AND #5 AND #6 AND #7 |
| 13 | #1 AND #2 AND #5 AND #6 AND #7 |
| 14 | #1 AND #3 AND #6 |
| 15 | #2 AND #3 AND #6 |
| 16 | #1 AND #4 |
